# Supplementary material for: Similar hemostatic responses to hypovolemia induced by hemorrhage and lower body negative pressure reveal a hyperfibrinolytic subset of non-human primates
Source: PLoS One. 2020 Jun 24;15(6):e0234844. doi: 10.1371/journal.pone.0234844 (PMC7314422; doi:10.1371/journal.pone.0234844)
Supplement: S1 File — (DOCX) [file pone.0234844.s001.docx]

**Supporting File 1: Animal Methods**

Animals: Adult male baboons (n=14) from the Southwest National Primate Research Center (SNRPC) were housed in individual cages in association with other baboons beginning 2 wk prior to experiments and ending 1 wk after study termination. The baboons were fed twice a day and given water ad libitum. Environmental enhancements in the form of structural, manipulable, and feeding enrichment were all available. Experiments were started when the baboons were accustomed to laboratory conditions. Animals received a prophylactic antibiotic (cefalozin iv) during all procedures. No significant pain was associated with either hemorrhage or LBNP. An anti-inflammatory agent (ketorolac) and stronger pain medications (tramadol or buprenorphine) were available at the discretion of the Responsible Veterinarian. Upon cessation of the study and following 1 week observation during recovery, the baboons were returned to their colony.

Complete information for the surgical procedures and for both the hemorrhage and LBNP studies were originally published in the *J Appl Physiol* 116: 406–415, 2014 (1); excerpted:

“The protocol was submitted to and approved by the Institutional Animal Care and Use Committee of the Texas Biomedical Research Institute, San Antonio, TX. The study was conducted in compliance with the Animal Welfare Act, the implementing Animal Welfare Regulations, and the principles of the Guide for the Care and Use of Laboratory Animals. Blood volume determination. Blood volume was determined in a separate group of male baboons (n=9) to prevent the need for an additional surgery and sedation procedure in animals exposed to hemorrhage and LBNP. Blood volume was calculated from the measurements of plasma volume with Evans blue dye technique (2) and hematocrit. Arterial blood samples were obtained before and 5 min after a bolus venous injection of Evans blue dye (Sigma) solution (0.5% in sterile saline, 1 ml/kg). Blood samples were analyzed to determine hematocrit by centrifugation, and plasma concentration of Evans blue by spectrophotometer compared with a standard curve.

Surgical procedures. On the day of each experimental session (hemorrhage or LBNP), the baboons were sedated with ketamine (10 mg/kg im). Sedation was maintained during the experiment with ketamine (10 mg⋅kg^-1^⋅h^-1^ iv) and valium (0.1mg ⋅kg^-1^⋅h^-1^ iv). The animals were intubated to maintain an open airway and allowed to breathe spontaneously during the experiments. For the hemorrhage and LBNP experiments, two vascular catheters were inserted using aseptic technique via small skin incisions to visualize the vessels. Arterial pressure and pulse pressure were measured via a pressure transducer attached to a Millar solid-state catheter inserted in the axillary artery. Central venous pressure (CVP) was measured via a pressure transducer attached to a fluid-filled catheter inserted in the axillary vein and advanced into the vena cava until the tip of the catheter was located proximal to the right atrium. Catheter tip placement was confirmed by assessing venous pressure waveforms for characteristics consistent with CVP. For hemorrhage experiments only, two additional catheters were inserted in the femoral artery and vein for blood removal and replacement, respectively. After catheter placement, ECG leads were attached to monitor heart rate. At the completion of the hemorrhage and LBNP experiments, the catheters were removed and the skin incisions at the catheter insertion sites were sutured closed.

Hemorrhage experiment. Blood pressure, heart rate, and CVP were recorded continuously during the experiment. Baseline values were monitored for 20 min prior to a stepwise hemorrhage of 25% blood volume calculated based on measurements of blood volume in a different group of baboons. The four steps of hemorrhage represented 6.25%, 12.5%, 18.75%, and 25% blood volume. Blood was removed by reversed infusion pump (50 ml/min) via the femoral artery, and each hemorrhage step was held for 7 min. Shed blood was collected into sterile citrate phosphate dextrose blood donation bags. Systolic arterial pressure was monitored closely during the hemorrhage procedure, and a value lower than 70 mmHg was considered an indicator of impending cardiovascular collapse. As an a priori protocol termination criterion, the hemorrhage procedure was stopped prematurely if this systolic arterial pressure threshold was attained prior to 25% blood loss. After the last step of hemorrhage, shed blood was replaced by infusion pump (50 ml/min) via the femoral vein. Hemodynamic variables were allowed to stabilize for 20 min prior to removing the catheters. Upon recovering from sedation, the animals were returned to their home cages and monitored for the resumption of normal feeding and drinking behavior.

Lower body negative pressure experiment (LBNP). Four weeks after the hemorrhage experiment, the baboons were again sedated and instrumented with axillary artery and vein catheters and ECG leads. The animals were placed supine in an airtight LBNP chamber sealed at the level of the iliac crest by a neoprene skirt. Blood pressure, heart rate, and CVP were recorded continuously during the experiment. Baseline values were monitored for 20 min prior to a stepwise LBNP procedure that was designed to match pulse pressure and CVP during the animal’s previous hemorrhage study. At the end of the procedure, the negative pressure was released, and hemodynamic variables were allowed to stabilize for 20 min. Catheters were removed, and the animals were allowed to recover from the effects of sedation before returning to their home cages.”

1. Hinojosa-Laborde C, Shade RE, Muniz GW, Bauer C, Goei KA, Pidcoke HF, et al. Validation of lower body negative pressure as an experimental model of hemorrhage. Journal Applied Physiology (1985). 2014;116(4):406-15.

2. Saunders JP, Horton RG, Weston RE. Blood volume in the dog determined by Evans blue and cyanide disappearance. Fed Proc. 1947;6(1 Pt 2):196.
