# Supplementary figures and images for: Similar hemostatic responses to hypovolemia induced by hemorrhage and lower body negative pressure reveal a hyperfibrinolytic subset of non-human primates
Source: PLoS One. 2020 Jun 24;15(6):e0234844. doi: 10.1371/journal.pone.0234844 (PMC7314422; doi:10.1371/journal.pone.0234844)

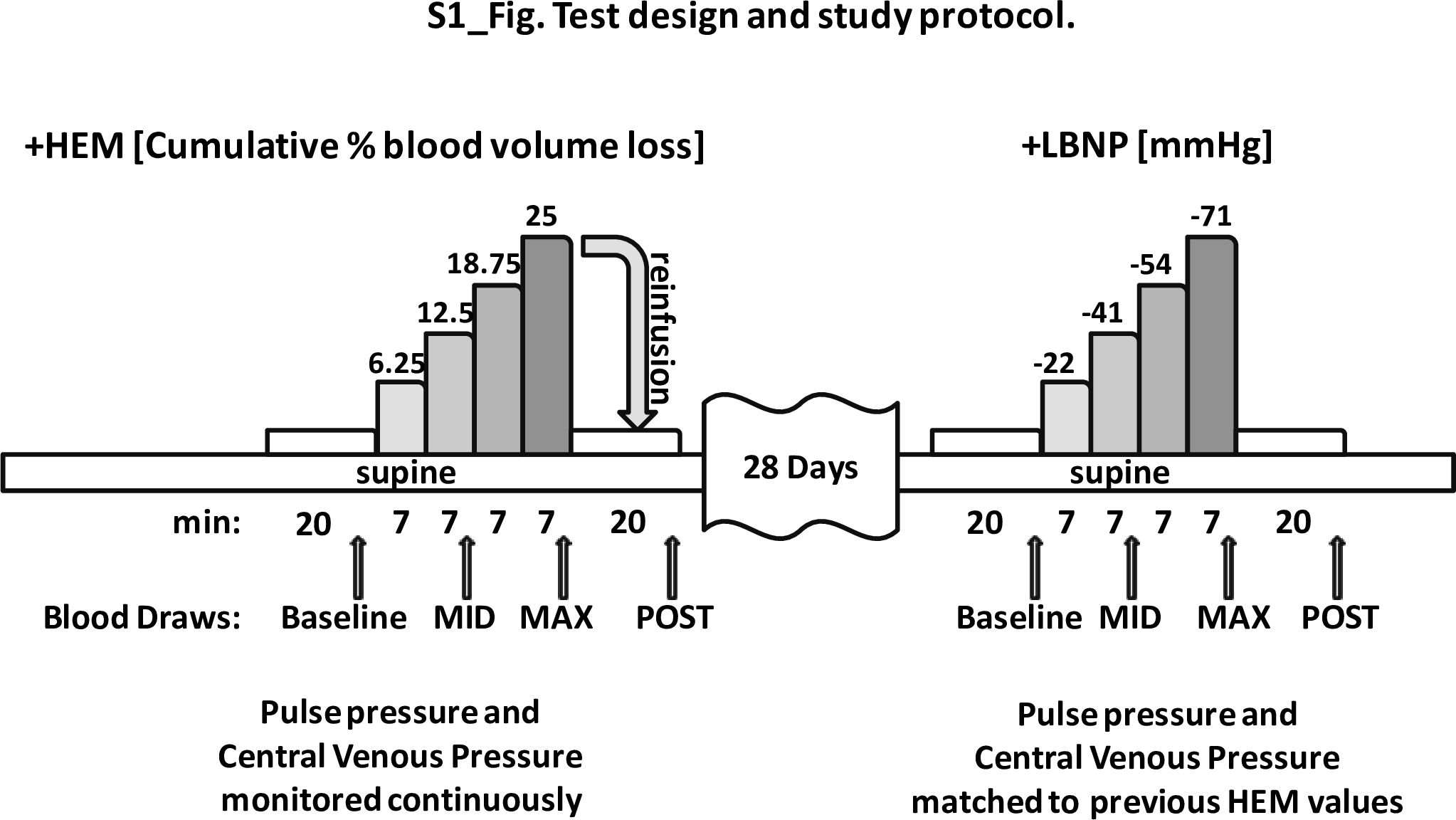

Supplement: S1 Fig — (TIF) [file pone.0234844.s002.tif]

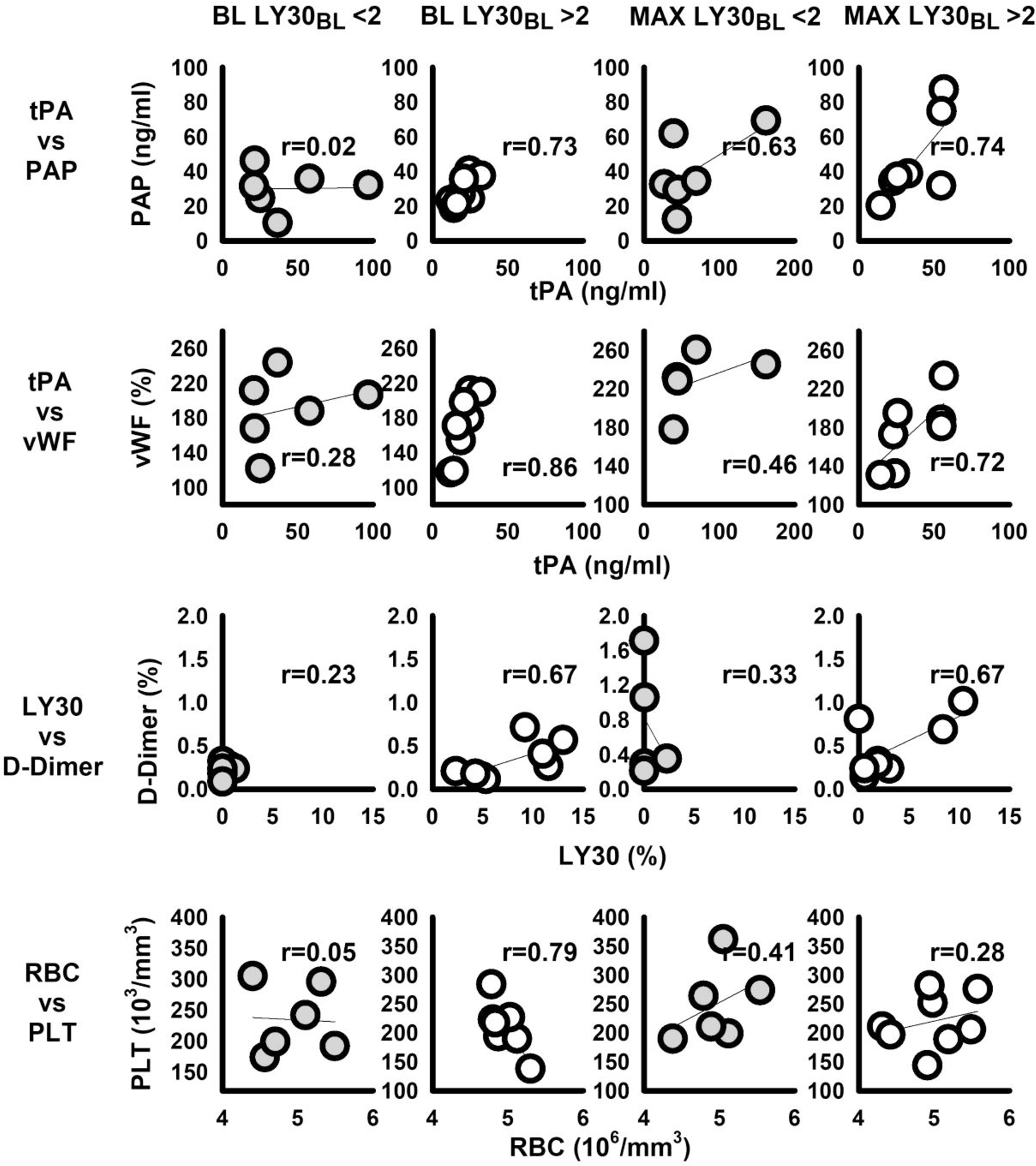

Supplement: S2 Fig — (TIF) [file pone.0234844.s003.tif]
